# Insights into human cardiac lipid dynamics through comprehensive arteriovenous lipidomics

**DOI:** 10.1016/j.jlr.2026.101152

**Published:** 2026-09-21

**Authors:** Lee Bockus, Nadia Chamoun, Ahmad Kassar, Yaacoub Chahine, Romanos Haykal, Jason Li, Dennis Wang, Kevin O’Brien, Danijel Djukovic, Daniel Raftery, Zoltan Arany, Rong Tian, Nona Sotoodehnia, Nazem Akoum

**Affiliations:** 1 Division of Cardiology, University of Washington, Seattle, WA, USA; 2 Northwest Metabolomics Research Center, Anesthesiology and Pain Medicine, University of Washington, Seattle, WA, USA; 3 Mitochondria and Metabolism Center, Department of Anesthesiology and Pain Medicine, University of Washington, Seattle, WA, USA; 4 Perelman School of Medicine, University of Pennsylvania, Philadelphia, PA, USA; 5 Department of Medicine, University of Washington, Seattle, WA, USA; 6 Department of Bioengineering, University of Washington, Seattle, WA, USA

**Keywords:** fatty acid/transport, lipolysis and fatty acid metabolism, glycerophospholipids, lysophospholipid, triglycerides, mitochondria

## Abstract

Free fatty acids (FFAs) are the adult heart’s primary energy substrate, but human cardiac lipid exchange remains incompletely characterized. We assessed cardiac lipid import and export using arteriovenous lipidomics in a prospective observational study of 59 fasting patients undergoing catheter ablation for atrial arrhythmias. Paired arterial and coronary sinus plasma samples underwent targeted lipidomics, detecting 1,464 lipid species across 20 classes; 851 species were analyzed. Paired comparisons used separate Benjamini–Hochberg corrections for classes and species. The heart showed net uptake of FFAs (−122.4 μmol/L; 95% confidence interval [CI], −146.0 to −98.8; q = 1.49 × 10⁻^13^) and nonsignificant triglyceride uptake. Lysophosphatidylcholine was released (+4.32 μmol/L; 95% CI, 2.45 to 6.19; q = 2.12 × 10⁻⁴); no other class met q < 0.05. Thirty-two species met q < 0.05. Release from other classes offset FFA and triglyceride uptake on average, yielding a positive aggregate acyl-chain-equivalent difference (+28.2 μmol acyl-chain equivalents/L), although the CI included both uptake and release (95% CI, −112.8 to 169.2; *P* = 0.690). This study characterizes distinct patterns of cardiac lipid exchange under fasting conditions and provides a basis for studies in heart failure and broader cardiometabolic disease. Further studies are warranted to identify the sources of released lipids and explore cardiac lipid handling in the fed state.

Lipid metabolism is integral to maintaining cardiac function, with FAs serving as the heart's primary energy source. Approximately 70% of the energy required for ATP production in cardiomyocytes is derived from FAs, with glucose, lactate, ketone bodies, and amino acids collectively contributing to the remainder (1, 2). The heart’s ability to tightly regulate lipid uptake and oxidation is essential not only as an energy source but also to prevent cardiomyocyte dysfunction and pathological remodeling from excess lipid accumulation (3, 4).

Animal studies have provided valuable insights into cardiac lipid metabolism, revealing adaptive mechanisms and pathological alterations (3). For instance, rabbit studies highlight hormone-regulated lipase activation (5). Rodent models have demonstrated dietary fat's impact on myocardial ceramide levels (6) and diabetes-induced changes in myocardial lipid composition (7). While these models provide valuable insights into regulatory mechanisms and metabolic shifts in response to stress, their physiological differences from humans limit their direct applicability.

In humans, cardiac metabolism has been studied using radiotracer techniques like positron emission tomography with radiolabeled substrates such as [^18^F]-fluorodeoxyglucose or [^11^C]-palmitate (8, 9, 10). These approaches are limited to single-compound studies and cannot capture the metabolome's complexity. Tissue metabolomics provides detailed profiles of intracellular metabolite levels (11, 12, 13); however, it primarily reflects the steady-state composition, limiting its ability to assess dynamic lipid exchange, or distinguish between active metabolism, storage or transport. Arteriovenous gradient analyses have successfully been used to assess metabolite exchange (14). In this study, we sought to characterize the human cardiac lipidomic profile through paired arteriovenous analyses, addressing the knowledge gap in understanding cardiac lipid dynamics.

## Materials and methods

### Study design and population

This observational study enrolled 59 patients undergoing catheter ablation for either atrial fibrillation (AF) or supraventricular tachycardia (SVT) at the University of Washington Medical Center (Seattle, WA) between 2021 and 2023. Patients with prior catheter ablation, an active or previous malignancy, a history of chemotherapy or radiation therapy, a left ventricular ejection fraction below 45%, or a heart rate ≥ 100 bpm were excluded. Subjects fasted overnight prior to the procedure. Venous samples were collected from the coronary sinus into sodium heparin phlebotomy tubes, while arterial samples were obtained from either the left atrium (in AF patients) or peripheral arteries (in SVT patients) (Fig. 1). All blood samples were obtained prior to the initiation of any catheter ablation or pacing interventions. AF patients were under general anesthesia and intubated with mechanical ventilation at the time of the blood sampling, while SVT patients were under moderate sedation. After blood draws, samples were placed on ice, followed by plasma isolation by refrigerated centrifugation and storage at −80°C. The study was conducted in accordance with the Declaration of Helsinki and was approved by the University of Washington Institutional Review Board (Human Research Protection Office, HSD#00014073). All participants provided written informed consent.

**Fig. 1:**
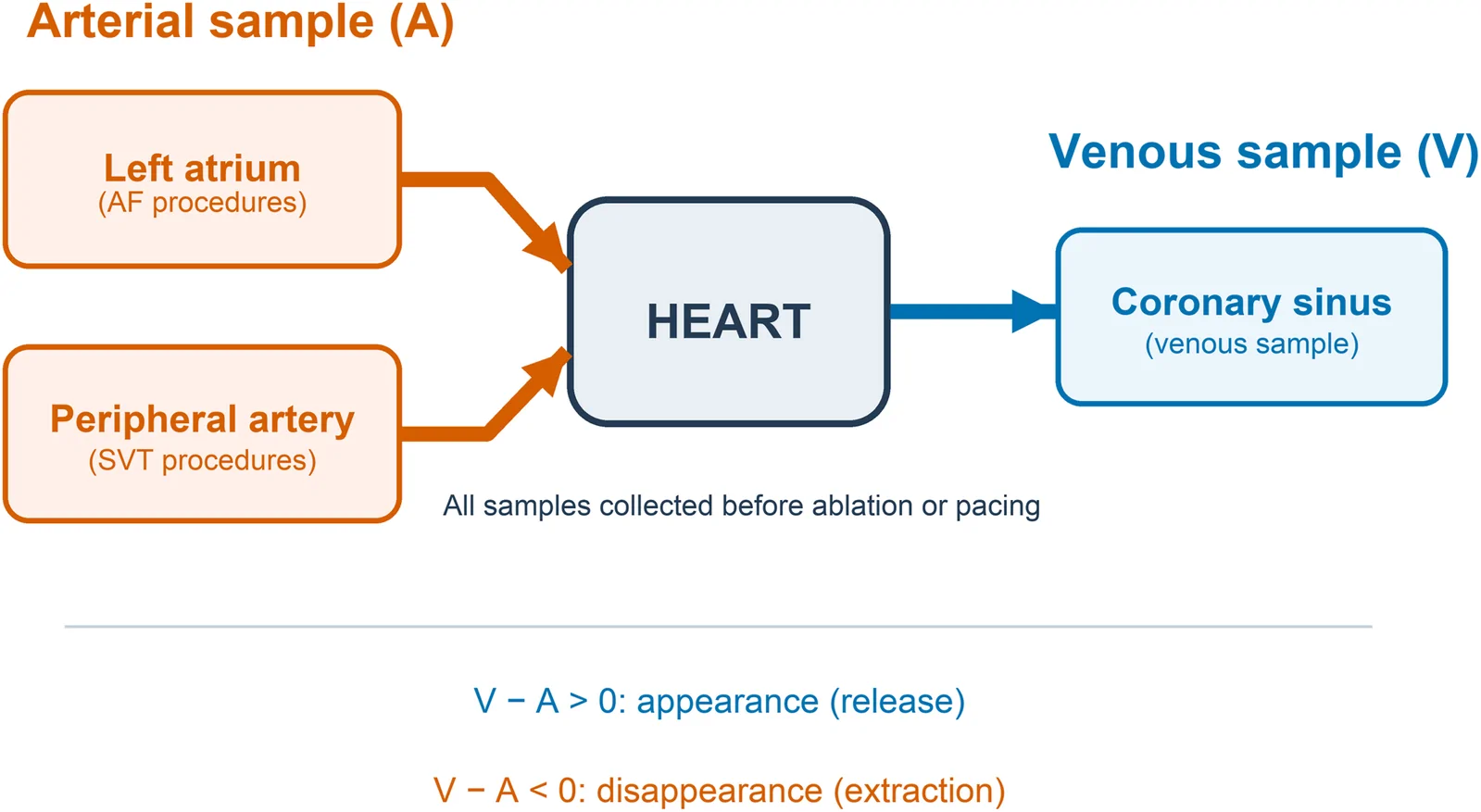
Venous and arterial sampling. Arterial blood (red arrows) was collected from either the left atrium or peripheral arteries, while venous blood (blue arrows) was drawn from the coronary sinus. The difference between venous and arterial concentrations (V–A) was calculated to assess net lipid exchange, where V−A >0 indicated net export and V−A <0 indicated net import. V−A, venous minus arterial concentration.

### Sample preparation of lipids

Samples were thawed at 4°C for 60 min and vortexed for 10 s. A 25-μL aliquot of each sample was transferred to a 2-mL Eppendorf PCR-clean vial. To this vial, 25 μl of a spiking solution containing 69 isotope-labeled lipid internal standards (Supplemental Lipidomics Data workbook, Sheet S9), 75 μl H_2_O, 575 μl methyl-tert-butyl ether, and 160 μl methanol were added. The mixture was vortexed, shaken at 21°C for 30 min, and centrifuged at 18,000 x g for 3 min. The upper layer was collected into a new glass tube.

The lower layer was reextracted with 300 μl methyl-tert-butyl ether, 100 μl methanol, and 100 μl H_2_O. This mixture was shaken and centrifuged again, with the upper layer combined with the previous extract. Samples were dried at 30°C using a Biotage N_2_ drier and reconstituted in 10 mM ammonium acetate in 50:50 methanol/methylene chloride for MS lipidomics analysis. Preparations were completed in batches of 32 samples, with MS data acquisition performed overnight.

### Mass spectrometry data acquisition

The Northwest Metabolomics Research Center conducted the lipidomic analyses. Lipid species were quantified using the SCIEX Lipidyzer platform, which integrates a Shimadzu Nexera X2 LC-30AD system, autosampler, and SCIEX QTRAP 5500 mass spectrometer equipped with an ESI source and SelexION differential mobility spectrometry (DMS). The assay targeted 1,572 unique multiple-reaction-monitoring (MRM) transitions. The assay does not use chromatography but relies on direct infusion. Each sample was analyzed in two method-specific injections, one with DMS on and one with DMS off. Data were processed using the open-source Shotgun Lipidomics Assistant software, yielding lipid concentrations (μmol/L).

The quantitative export contained 1,561 species columns; 1,464 had at least one numeric measurement, 1,226 had at least two complete arterial–venous pairs, and 851 met the missingness criterion for species analysis. Supplemental Sheets S7–S9 reconcile these counts and provide Q1 precursor m/z, Q3 product-ion m/z, polarity, reference ion-form/adduct and DMS assignments, and all 69 isotope-labeled internal standards with their spike concentrations. Reference assignments were obtained by matching target name, Q1, Q3, and polarity to the public Shotgun Lipidomics Assistant KeyV4 v1.3 dictionary (15) (https://sciex.com/content/dam/SCIEX/tech-notes/life-science-research/lipidomics/ruo-mkt-02-14475-a/SCIEX-Targeted-Method_FIA-Lipidomics_6500_5500__RUO-MKT-02-14428-A.pdf) (https://github.com/syjgino/SLA/blob/v1.3-keyV4/doc/KeyV4/spname_dict_V4_1.3_20221012.xlsx); all 1,572 transitions matched. FA transitions with Q1 = Q3 are identified as pseudo-MRM.

Samples were analyzed in five daily batches, each with a prepared blank and internal QC serum at the beginning and end. Median intraday CVs ranged from 6.2% to 7.5%; among 887 species quantified in all 10 QC injections, the median interday CV was 10.1%. Additional method and quality-control details are provided in the completed LSI checklist and supplemental workbook.

### Statistical analysis

Continuous variables are reported as mean ± SD or as median and interquartile range. Categorical variables are summarized as available-case counts and percentages. The participant was the analysis unit. Within-person differences were calculated as coronary sinus venous minus arterial concentration (V−A); negative values indicate extraction and positive values indicate release. Blanks, nonnumeric entries, and zeros were treated as missing, and no values were imputed. Species with no more than 10% missing among the 118 measurements were included (851 species; paired n = 48–59).

Class and species V−A differences were assessed using two-sided paired t tests, with Wilcoxon signed-rank tests as sensitivity analyses. Benjamini–Hochberg false-discovery-rate correction was applied separately to the 20 class and 851 species tests; q < 0.05 was considered significant. Results are reported as paired n, arterial and venous concentrations, mean V−A with 95% CI, Cohen d_z_, and within-participant percentage change when the arterial concentration was available and greater than zero. Raw *P* values are descriptive when q ≥ 0.05.

As a secondary analysis, each participant’s class-level V−A difference was multiplied by a class-specific acyl-chain multiplier: one for FFA, CE, Cer, HexCer, LacCer, SM, LPC, LPE, LPG, LPI, and LPS; two for DG, PA, PC, PE, PG, PI, and PS; and three for TAG. The contributions were summed within participant to give an aggregate acyl-chain-equivalent difference and tested against zero using a one-sample *t* test and a Wilcoxon signed-rank test. This concentration-based estimate is not carbon balance or organ flux because coronary blood flow and intracellular metabolism were not measured.

For descriptive reconciliation, the mean contributions of the 851 included species were also summed; each fatty-acyl-fragment-resolved TAG species was counted once. This was not treated as an inferential endpoint because paired n differed among species. Analyses were performed in R version 4.2.1. Complete class and species statistics, deidentified concentrations, missingness, QC summaries, aggregate calculations, transitions, and internal-standard information are provided in the supplemental workbook.

## Results

### Baseline characteristics

The study cohort (Table 1) consisted of 59 participants with a median age of 70 years (IQR: 59–73). The cohort was predominantly male (55.9%), with a mean BMI of 28.9 ± 5.7 kg/m^2^. At the time of presentation and blood sampling, 33.9% of patients were in AF, while the remaining were in sinus rhythm. Among those in sinus rhythm, 33 had a history of AF, and six had a history of SVT. The prevalence of comorbidities included hypertension in 29 of 58 participants (50.0%), coronary artery disease in 7 of 42 (16.7%), and diabetes mellitus in 3 of 59 (5.1%). In addition, 69.5% of participants were on beta-blockers, 32.2% on antiarrhythmic agents, and 22.0% on calcium channel blockers. The cohort had a left ventricular ejection fraction of 62.4 ± 6.2%, as determined from preprocedural echocardiograms in 53 participants. Lipid-lowering therapy was recorded for 25 of 59 participants (42.4%): 23 (39.0%) received a statin, two (3.4%) ezetimibe, and one (1.7%) evolocumab. Medication classes were not mutually exclusive, and treatment timing relative to sampling was unavailable.

**Table 1:** Baseline characteristics of the study cohort

Characteristic	Value
Age, years, median (IQR)	70.0 (59–73)
Male sex, n/N (%)	33/59 (55.9)
Female sex, n/N (%)	26/59 (44.1)
BMI, kg/m^2^, mean ± SD	28.9 ± 5.7
Rhythm: SVT in NSR, n/N (%)	6/59 (10.2)
Rhythm: AF in NSR, n/N (%)	33/59 (55.9)
Rhythm: AF in AF, n/N (%)	20/59 (33.9)
Diabetes mellitus, n/N (%)	3/59 (5.1)
Hypertension, n/N (%)	29/58 (50.0)
Valvular disease, n/N (%)	5/59 (8.5)
Coronary artery disease, n/N (%)	7/42 (16.7)
Beta blocker, n/N (%)	41/59 (69.5)
Calcium channel blocker, n/N (%)	13/59 (22.0)
Antiarrhythmic drug, n/N (%)	19/59 (32.2)
Left ventricular ejection fraction,%, mean ± SD	62.4 ± 6.2 (N = 53)
Any recorded lipid-lowering therapy, n/N (%)	25/59 (42.4)
Statin therapy, n/N (%)	23/59 (39.0)
Ezetimibe therapy, n/N (%)	2/59 (3.4)
PCSK9 inhibitor therapy, n/N (%)	1/59 (1.7)

Values are n/N (%) unless noted; percentages use available-case denominators. Missing: hypertension, n = 1; coronary artery disease, n = 17; left ventricular ejection fraction, n = 6. Lipid-lowering classes are not mutually exclusive, and medication timing was unavailable.

AF, atrial fibrillation; IQR, interquartile range; NSR, normal sinus rhythm; PCSK9, proprotein convertase subtilisin/kexin type 9; SVT, supraventricular tachycardia.

### Arterial concentrations of lipid metabolites

Arterial lipid concentrations were measured and are detailed in the supplemental workbook and Figure 2. FFA showed a mean concentration of 1,095 ± 310 μmol/L, and cholesteryl esters presented a mean of 2,920 ± 693 μmol/L. Other notable lipid classes included triacylglycerol (TAG) at 812 ± 548 μmol/L and phosphatidylcholine (PC) at 1,768 ± 431 μmol/L.

**Fig. 2:**
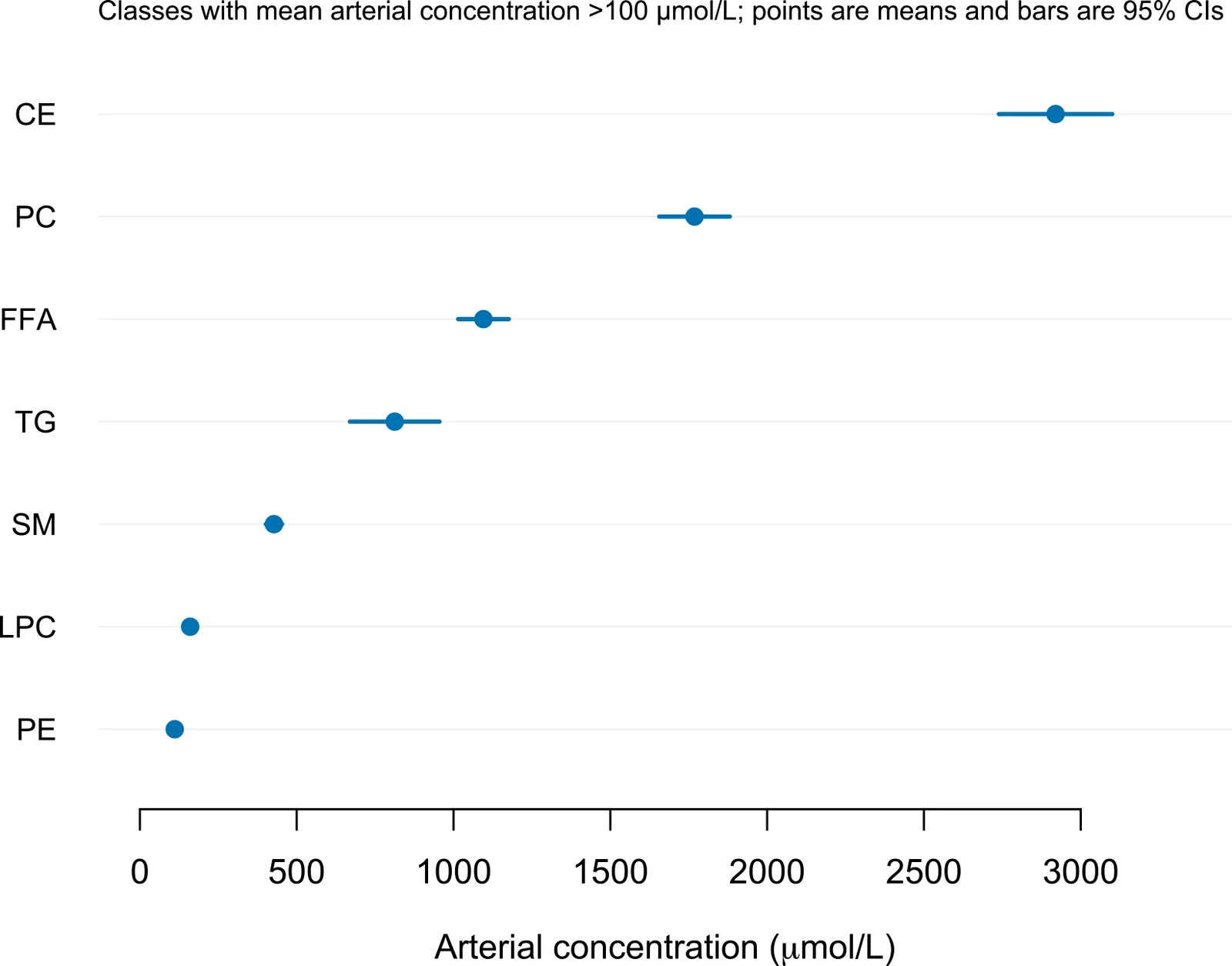
Arterial concentrations of lipids. Forest plot depicting arterial concentrations (mean arterial concentration >100 μmol/L) of lipids in the cohort of 59 patients. Means and 95% confidence intervals (CIs) are shown.

### Venous-arterial differences in lipid metabolite classes

The V−A differences (Fig. 3A and the supplemental workbook) and percentage changes together highlight the dynamics of cardiac lipid handling. FFAs were the main lipid imported (mean V−A, −122.4 ± 90.5 μmol/L; 95% CI, −146.0 to −98.8; d_z_ = −1.35; mean percentage change, −11.1%; *P* = 7.4 × 10^−15^; q = 1.49 × 10^−13^). Lysophosphatidylcholine showed a smaller release signal (+4.32 ± 7.17 μmol/L; 95% CI, 2.45 to 6.19; d_z_ = 0.60; mean percentage change, +2.94%; *P* = 2.12 × 10^−5^; q = 2.12 × 10^−4^). These were the only class-level findings with q < 0.05 (Fig. 3A). Phosphatidylcholine, cholesterol ester, phosphatidylethanolamine, phosphatidylglycerol, sphingomyelin, lysophosphatidylglycerol, and lysophosphatidylinositol had nominal *P* < 0.05 but did not survive correction across the 20 classes.

**Fig. 3:**
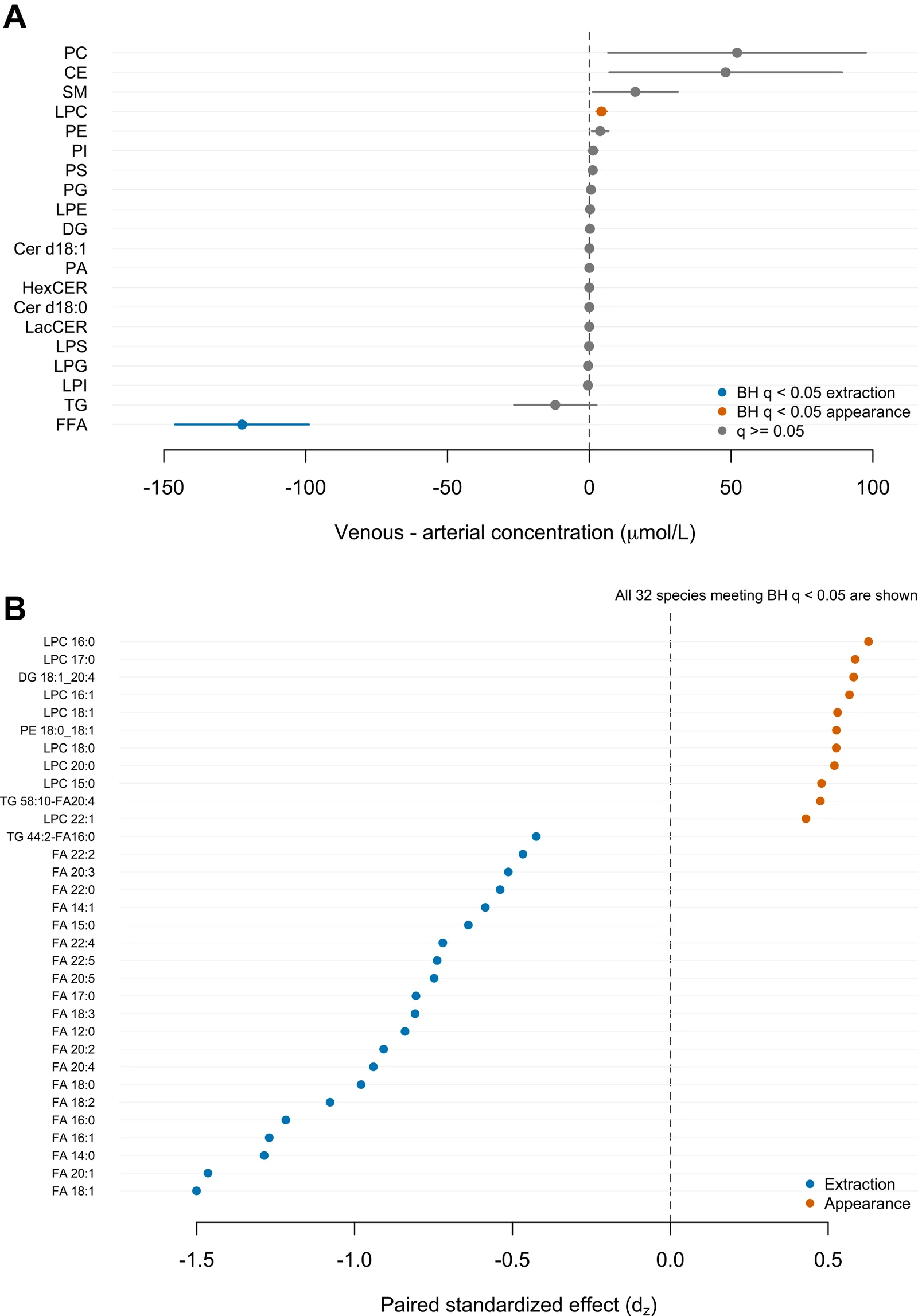
Differences in venous versus arterial lipid concentrations. A: Mean venous–arterial differences and 95% CIs for all 20 lipid classes. B: Paired standardized effect sizes (d_z_) for the 32 of 851 species meeting Benjamini–Hochberg q < 0.05. Negative values indicate extraction and positive values indicate release. Blue and orange denote significant extraction and release, respectively; gray denotes q ≥ 0.05. LPC, lysophosphatidylcholine.

TAG showed a smaller, nonsignificant mean uptake of −12.0 ± 55.7 μmol/L (95% CI, −26.5 to 2.5; d_z_ = −0.22; mean percentage change, −0.71%; *P* = 0.104; q = 0.185). Contemporaneous conventional serum triglyceride and FFA measurements were not available; quantitative class and species results from the targeted assay are therefore reported directly.

At the species level, 32 of 851 tested species met q < 0.05 (Fig. 3B and the supplemental workbook). These included 20 imported free-fatty-acid species, eight exported lysophosphatidylcholine species, one exported phosphatidylethanolamine species, one exported diacylglycerol species, and two TAG species that changed in opposite directions. The largest absolute differences were FA 18:1 (−50.0 μmol/L), FA 18:2 (−25.9 μmol/L), FA 16:0 (−23.8 μmol/L), FA 18:0 (−6.59 μmol/L), and FA 16:1 (−5.81 μmol/L), all with q < 0.05. No phosphatidylcholine or cholesterol-ester species survived species-level correction.

Four odd-chain species met q < 0.05. FA 15:0 and FA 17:0 were taken up, whereas LPC 15:0 and LPC 17:0 were released. Dietary intake and endogenous fatty-acid metabolism may both contribute, but their source cannot be determined from these data (16, 17).

The participant-level aggregate acyl-chain-equivalent V−A difference averaged +28.2 ± 541.0 μmol acyl-chain equivalents/L (95% CI, −112.8 to 169.2; one-sample *t* test *P* = 0.690; Wilcoxon *P* = 0.711; Fig. 4A). On average, the positive point estimate reflected release from other classes that more than offset the negative free-fatty-acid and TAG contributions, although the wide confidence interval included both net uptake and net release. Figure 4B shows the contribution and acyl-chain multiplier for each class.

**Fig. 4:**
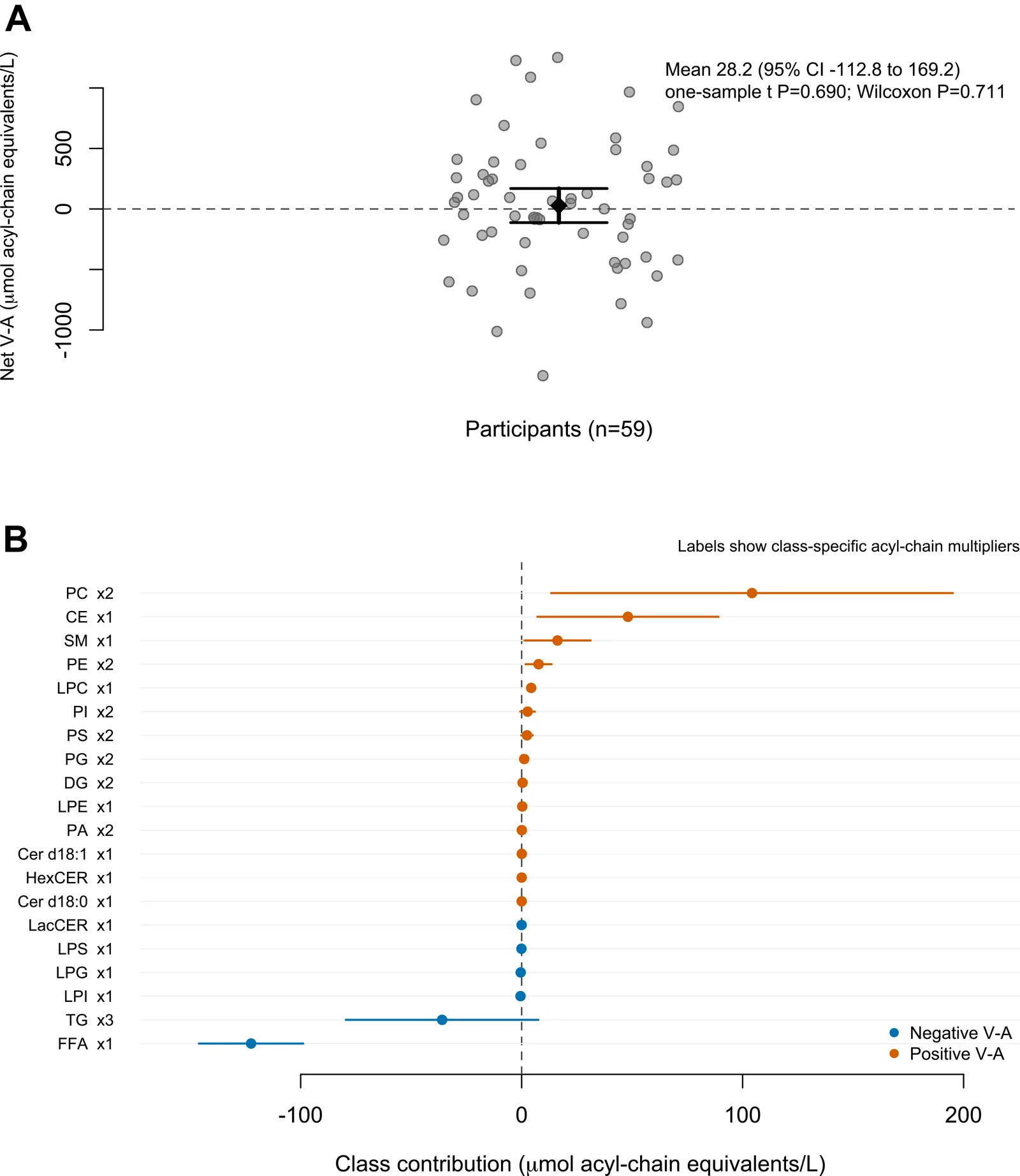
Aggregate venous–arterial acyl-chain-equivalent differences. A: Participant-level differences (n = 59), with the mean and 95% CI. *P* values are from a one-sample *t* test and Wilcoxon signed-rank test. B: Mean contribution and 95% CI for each lipid class after applying the indicated acyl-chain multiplier. Negative values indicate extraction and positive values indicate release.

The sum of the 851 species-level mean contributions was +27.2 μmol acyl-chain equivalents/L, closely matching the +28.2 μmol acyl-chain equivalents/L participant-level class estimate. This comparison was descriptive because complete-pair sample sizes differed among species.

## Discussion

This study of 59 fasting subjects provides a detailed assessment of human cardiac lipid import and export. Consistent with prior research by Murashige *et al.* (14), the heart exhibited net uptake of FFAs, aligning with its established energy demands, while simultaneously releasing selected lipids. Lysophosphatidylcholine release and 32 species-level differences remained significant after correction for multiple testing. The aggregate acyl-chain-equivalent point estimate was positive because release from other classes offset FFA and triglyceride uptake on average, although the confidence interval included both uptake and release.

As expected, FFAs were the predominant lipid imported by the heart, aligning with previous research that identifies them as the primary energy substrate for cardiomyocytes (14, 18, 19). Upon entering cardiomyocytes, FFAs are largely oxidized in mitochondria, with a smaller fraction incorporated into structural lipids or stored as triglycerides (9, 20, 21). The pattern of FFA uptake extended across 20 species, led by FA 18:1, FA 18:2, and FA 16:0. Beyond their role in energy metabolism, FFAs also contribute to intracellular signaling, gene regulation, and posttranslational modifications, emphasizing their multifaceted roles in cardiac function (22, 23). TAG showed a smaller and more variable uptake estimate, and the two significant TAG species changed in opposite directions.

Lysophosphatidylcholine release was modest in absolute and relative magnitude but remained significant after correction for multiple testing. Eight lysophosphatidylcholine species contributed to this pattern, led by LPC 16:0. This finding may reflect phospholipid remodeling or exchange, but an arteriovenous concentration difference cannot distinguish synthesis, hydrolysis, cellular release, or altered clearance.

The four odd-chain findings changed in opposite directions: FA 15:0 and FA 17:0 were taken up, whereas LPC 15:0 and LPC 17:0 were released. Odd-chain FAs may reflect dietary intake or endogenous metabolism (16, 17), but the present platform cannot determine their source. These findings require confirmation before a specific pathway can be assigned.

Taken together, these results reveal an underappreciated role of the heart in exporting selected lipids under fasting conditions, while still taking up FFAs as a primary energy source.

### Limitations

A limitation of relying on arteriovenous differences in this human population is the clinical necessity of the fasting state and sedation or anesthesia for the catheter ablation procedure. Patients with AF received general anesthesia and mechanical ventilation, whereas those with SVT received moderate sedation. Thus, the metabolic context may not fully represent typical physiological conditions, and the use of propofol during anesthesia might alter circulating lipid levels. While arteriovenous sampling offers a valuable window into net substrate exchange, concentration differences do not measure organ flux without coronary blood-flow measurements and do not provide information on intracellular pathways or the precise mechanisms driving uptake and export. Furthermore, the modest size of this cohort limits statistical power, so certain conclusions must remain hypothesis-generating until validated in a larger cohort. The heterogeneity of the patient population, including individuals with coronary artery disease and the use of medications such as beta-blockers, calcium channel blockers, and lipid-lowering therapy, may influence myocardial energetics and substrate utilization. Medication timing relative to sampling was unavailable. The findings may also reflect age-related changes in the older study population and may not generalize to younger or healthy hearts. This cohort reflects a real-world population undergoing catheter ablation for atrial arrhythmias, enhancing the clinical relevance of our findings, but also limiting generalizability.

## Conclusion

In summary, this comprehensive assessment of human cardiac lipid dynamics via arteriovenous lipidomics reveals simultaneous lipid import and export during fasting, with FFAs the predominant lipid imported. FFA uptake and release of selected lipids demonstrate distinct handling across lipid classes and molecular species. Further studies are warranted to clarify the source of the released lipids and to explore cardiac lipid handling in the fed state.

## Data availability

Deidentified processed class- and species-level concentrations, complete statistical results, MRM transitions, internal-standard information, and QC summaries are provided in the supplemental workbook. The original mass-spectrometry acquisition files are archived by the core laboratory but have not been deposited in a public repository. Requests for access may be directed to Nazem Akoum (University of Washington; nakoum@uw.edu), subject to deidentification and institutional approval.

## Supplemental data

This article contains supplemental data.

## Declaration of Generative AI and AI-Assisted Technologies in the Writing Process

During preparation of the revised manuscript, the authors used OpenAI Codex for language editing and internal-consistency checking. The authors reviewed and edited the content as needed and take full responsibility for the content of the published article.

## Conflict of interest

The authors declare that they have no conflicts of interest with the contents of this article.

## Supplemental data

Supplemental Material

LSI Checklist
