# Supplementary material for: Insights into human cardiac lipid dynamics through comprehensive arteriovenous lipidomics
Source: J Lipid Res. 2026 Sep 21;67(10):101152. doi: 10.1016/j.jlr.2026.101152 (PMC13696132; doi:10.1016/j.jlr.2026.101152)
Supplement: Supplementary file 1 [file mmc2.pdf]

Created by

Contents of Report https://lipidomicstandards.org, version v2.6.1

Direct Infusion Workflow 1
Overall study design 1
Lipid extraction 2
Analytical platform 2
Quality control 2
Method qualification and validation 2
Reporting 2

Sample Descriptions 3
Paired arterial and coronary-sinus plasma (59 arterial, 59 venous) / Human / Plasma 3

Lipid Class Descriptions 3
1) CE[M+NH4]+ / Lipid identification 3
1) CE[M+NH4]+ / Lipid quantification 4
2) Cer[M+H]+ / Lipid identification 5
2) Cer[M+H]+ / Lipid quantification 5
3) DG[M+NH4]+ / Lipid identification 6
3) DG[M+NH4]+ / Lipid quantification 6
4) FA[M-H]- / Lipid identification 7
4) FA[M-H]- / Lipid quantification 8
5) HexCer[M+H]+ / Lipid identification 8
5) HexCer[M+H]+ / Lipid quantification 9
6) LPC[M+H]+ / Lipid identification 9
6) LPC[M+H]+ / Lipid quantification 10
7) LPE[M+H]+ / Lipid identification 10
7) LPE[M+H]+ / Lipid quantification 11
8) LPG[M+NH4]+ / Lipid identification 11
8) LPG[M+NH4]+ / Lipid quantification 12
9) LPI[M+NH4]+ / Lipid identification 13
9) LPI[M+NH4]+ / Lipid quantification 13
10) LPS[M+H]+ / Lipid identification 14
10) LPS[M+H]+ / Lipid quantification 14
11) LacCer[M+H]+ / Lipid identification 15
11) LacCer[M+H]+ / Lipid quantification 16
12) PA[M+NH4]+ / Lipid identification 16
12) PA[M+NH4]+ / Lipid quantification 17
13) PC[M+CH3COO]- / Lipid identification 17
13) PC[M+CH3COO]- / Lipid quantification 18
14) PE[M-H]- / Lipid identification 18
14) PE[M-H]- / Lipid quantification 19
15) PG[M-H]- / Lipid identification 19
15) PG[M-H]- / Lipid quantification 20
16) PI[M-H]- / Lipid identification 21
16) PI[M-H]- / Lipid quantification 21
17) PS[M-H]- / Lipid identification 22
17) PS[M-H]- / Lipid quantification 22
18) SM[M+H]+ / Lipid identification 23
18) SM[M+H]+ / Lipid quantification 24
19) TG[M+NH4]+ / Lipid identification 24
19) TG[M+NH4]+ / Lipid quantification 25

Direct Infusion Workflow

Overall study design

Title of the study	Insights into Human Cardiac Lipid Dynamics Through Comprehensive Arteriovenous Lipidomics		
Document creation date	09/07/2026	Principal investigator	Daniel Raftery, PhD; Northwest Metabolomics Research Center
Institution	University of Washington	Corresponding Email	leebo@uw.edu
Is the workflow targeted or untargeted?	Targeted	Clinical	Yes

Lipid extraction

Extraction method	2-phase system	pH adjustment	None
2-phase system	MTBE	Special conditions	25 µL plasma plus 25 µL of the 69-standard spike; MTBE/methanol/water extraction with lower-phase re-extraction, nitrogen drying and ammonium-acetate reconstitution.
Derivatization	None in the documented protocol.	Were internal standards added prior extraction?	Yes

Analytical platform

Ionizaton additives	Ammonium acetate	Detector	Mass spectrometer
MS type	QTrap	MS vendor	SCIEX
Direct type	FIA	MS Level	MS^2
Mass window for precursor ion isolation (in Da total isolation window)	0.7	Mass resolution for detected ion at MS^2	Low resolution
Resolution at MS^2	Unit	Recording mode of raw data at MS^2	Centroid mode
Was/Were additional dimension/techniques used	Yes		

Quality control

Blanks	Yes	Type of Blanks	Extraction blank
Quality control	Yes	Type of QC sample	Sample pool

Method qualification and validation

Method validation	Yes	Lipid recovery	No
Dynamic quantification range	No	Limit of quantitation (LOQ)/Limit of detection (LOD)	No
Precision	Yes	Accuracy	No
Guidelines followed	Unknown; no validation guideline was specified in the retained records.		

Reporting

Are reported raw data uploaded into repository?	No	Are metadata available?	Yes

Raw data upload	No	Additional comments	The cohort comprises 59 arterial/coronary-sinus pairs (118 plasma samples). Samples were kept on ice, centrifuged under refrigeration and stored at -80°C; exact processing times are unknown. Unit resolution and a nominal 0.7-Da FWHM isolation width are inferred from the matching SCIEX workflow, not recovered settings. Centroid is the closest category for discrete MRM data; the original acquisition flag is unknown. Extraction blanks and pooled laboratory QC serum are inferred from five prepared blanks and ten internal QC-serum measurements. The serum is not assumed to be a pool of study plasma; exact control preparation/source is unknown. No selections for recovery, dynamic range, LOQ and accuracy provisionally assume no separate study-specific evaluation. These assumptions are unverified; precision was evaluated using QC samples. Correction and standard-verification assumptions are stated in the class remarks. Processed concentrations, transitions, standards and QC results are provided in Supplemental_Lipidomics_Data.xlsx. Raw files remain archived at the core but were not publicly deposited or inspected for this revision; access inquiries: nakoum@uw.edu, subject to deidentification and institutional approval.

Sample Descriptions

Paired arterial and coronary-sinus plasma (59 arterial, 59 venous) / Human / Plasma

Storage and collection conditions	Available	Provided preanalytical information	Preservation method
Temperature handling original sample	Unknown	Instant sample preparation	No
Storage temperature	-80 °C	Additives	Sodium-heparin anticoagulant in blood collection tubes

Lipid Class Descriptions

1) CE[M+NH4]+ / Lipid identification

Lipid class	CE	MS Level for identification	MS^2
Identification level	Species level	MS^2 adduct	[M+NH4]+
Fragments for identification			

Fragment name
Product-ion m/z; named fragment not retained (see Supplemental Sheet S8)

Isotope correction at MS^2	Type 2	MS^2 verified by standard	Yes
Background check at MS^2	Yes	Did you presume assumptions for identification?	Yes
Which assumptions were presumed?	Assignments follow the predefined MRM panel at the reported structural level; positional isomers are not established.	Limit of detection	Unknown; study-specific LOD criteria were not retained.
Additional dimension/techniques	SelexION differential mobility spectrometry (DMS)	How was/were the additional dimension(s) used?	Public platform reference: DMS transparent/off; study-specific settings were not retained
Was a model used to predict lipid molecule separation?	No	Lipid Identification Software	Shotgun Lipidomics Assistant (version not retained)
Nomenclature for intact lipid molecule	Yes	Nomenclature for fragment ions	N/A
Further identification remarks	Type II correction is assumed from the matched public SLA v1.3 workflow; study-specific settings are unknown. Class-level product-ion checking is inferred from the listed isotope standards; an independent verification experiment is not documented. Ion forms and DMS assignments are public-workflow references (Sheet S8). Background checking denotes prepared-blank monitoring; interference criteria are unknown.		

1) CE[M+NH4]+ / Lipid quantification

Quantitative	Yes	MS Level for quantification	MS^2

Internal lipid standard(s) MS^2

Internal standard	Fragment(s)	Endogenous subclass
5 class-matched isotope-labeled standards; see Supplemental Sheet S9	Product-ion m/z; name not retained	Analyte-to-standard assignment not retained

Type of quantification	Internal standard amount	Response correction	No
Type I isotope correction	Yes	Limit of quantification	Unknown; study-specific LOQ criteria were not retained.
Normalization to reference	Yes	Lipid Quantification Software	Shotgun Lipidomics Assistant (version not retained)
Batch correction	Original batch correction is unknown. No further batch correction was applied to the supplied concentrations during revision.	Further quantification remarks	Internal-standard quantification, plasma-volume normalization and Type I correction are inferred from the matched SLA workflow. No separate species-response model is assumed beyond internal-standard normalization. Original settings and analyte-to-standard assignments are unknown. No additional correction of supplied concentrations was applied during revision.

2) Cer[M+H]+ / Lipid identification

Lipid class	Cer	MS Level for identification	MS^2
Identification level	Molecular species level	MS^2 adduct	[M+H]+

Fragments for identification

Fragment name
Product-ion m/z; named fragment not retained (see Supplemental Sheet S8)

Isotope correction at MS^2	Type 2	MS^2 verified by standard	Yes
Background check at MS^2	Yes	Did you presume assumptions for identification?	Yes
Which assumptions were presumed?	Assignments follow the predefined MRM panel at the reported structural level; positional isomers are not established.	Limit of detection	Unknown; study-specific LOD criteria were not retained.
Additional dimension/techniques	SelexION differential mobility spectrometry (DMS)	How was/were the additional dimension(s) used?	Public platform reference: DMS transparent/off; study-specific settings were not retained
Was a model used to predict lipid molecule separation?	No	Lipid Identification Software	Shotgun Lipidomics Assistant (version not retained)
Nomenclature for intact lipid molecule	Yes	Nomenclature for fragment ions	N/A
Further identification remarks	Type II correction is assumed from the matched public SLA v1.3 workflow; study-specific settings are unknown. Class-level product-ion checking is inferred from the listed isotope standards; an independent verification experiment is not documented. Verification of the d18:0 subgroup is not established. Ion forms and DMS assignments are public-workflow references (Sheet S8). Background checking denotes prepared-blank monitoring; interference criteria are unknown.		

2) Cer[M+H]+ / Lipid quantification

Quantitative	Yes	MS Level for quantification	MS^2

Internal lipid standard(s) MS^2

Internal standard	Fragment(s)	Endogenous subclass
5 class-matched isotope-labeled standards; see Supplemental Sheet S9	Product-ion m/z; name not retained	Analyte-to-standard assignment not retained

Type of quantification	Internal standard amount	Response correction	No
Type I isotope correction	Yes	Limit of quantification	Unknown; study-specific LOQ criteria were not retained.
Normalization to reference	Yes	Lipid Quantification Software	Shotgun Lipidomics Assistant (version not retained)

Batch correction	Original batch correction is unknown. No further batch correction was applied to the supplied concentrations during revision.	Further quantification remarks	Internal-standard quantification, plasma-volume normalization and Type I correction are inferred from the matched SLA workflow. No separate species-response model is assumed beyond internal-standard normalization. Original settings and analyte-to-standard assignments are unknown. No additional correction of supplied concentrations was applied during revision.

3) DG[M+NH4]+ / Lipid identification

Lipid class	DG	MS Level for identification	MS^2
Identification level	Molecular species level	MS^2 adduct	[M+NH4]+
Fragments for identification			

Fragment name
Product-ion m/z; named fragment not retained (see Supplemental Sheet S8)

Isotope correction at MS^2	Type 2	MS^2 verified by standard	Yes
Background check at MS^2	Yes	Did you presume assumptions for identification?	Yes
Which assumptions were presumed?	Assignments follow the predefined MRM panel at the reported structural level; positional isomers are not established.	Limit of detection	Unknown; study-specific LOD criteria were not retained.
Additional dimension/techniques	SelexION differential mobility spectrometry (DMS)	How was/were the additional dimension(s) used?	Public platform reference: DMS transparent/off; study-specific settings were not retained
Was a model used to predict lipid molecule separation?	No	Lipid Identification Software	Shotgun Lipidomics Assistant (version not retained)
Nomenclature for intact lipid molecule	Yes	Nomenclature for fragment ions	N/A
Further identification remarks	Type II correction is assumed from the matched public SLA v1.3 workflow; study-specific settings are unknown. Class-level product-ion checking is inferred from the listed isotope standards; an independent verification experiment is not documented. Ion forms and DMS assignments are public-workflow references (Sheet S8). Background checking denotes prepared-blank monitoring; interference criteria are unknown.		

3) DG[M+NH4]+ / Lipid quantification

Quantitative	Yes	MS Level for quantification	MS^2
Internal lipid standard(s) MS^2			

Internal standard	Fragment(s)	Endogenous subclass

5 class-matched isotope-labeled standards; see Supplemental Sheet S9	Product-ion m/z; name not retained	Analyte-to-standard assignment not retained

Type of quantification	Internal standard amount	Response correction	No
Type I isotope correction	Yes	Limit of quantification	Unknown; study-specific LOQ criteria were not retained.
Normalization to reference	Yes	Lipid Quantification Software	Shotgun Lipidomics Assistant (version not retained)
Batch correction	Original batch correction is unknown. No further batch correction was applied to the supplied concentrations during revision.	Further quantification remarks	Internal-standard quantification, plasma-volume normalization and Type I correction are inferred from the matched SLA workflow. No separate species-response model is assumed beyond internal-standard normalization. Original settings and analyte-to-standard assignments are unknown. No additional correction of supplied concentrations was applied during revision.

4) FA[M-H]- / Lipid identification

Lipid class	FA	MS Level for identification	MS^2
Identification level	Species level	MS^2 adduct	[M-H]-

Fragments for identification

Fragment name
No fragment; Q1=Q3 pseudo-MRM monitors the intact fatty-acid ion (Sheet S8).

Isotope correction at MS^2	Type 2	MS^2 verified by standard	No
Background check at MS^2	Yes	Did you presume assumptions for identification?	Yes
Which assumptions were presumed?	Assignments follow the predefined MRM panel at the reported structural level; positional isomers are not established.	Limit of detection	Unknown; study-specific LOD criteria were not retained.
Additional dimension/techniques	SelexION differential mobility spectrometry (DMS)	How was/were the additional dimension(s) used?	Public platform reference: DMS transparent/off; study-specific settings were not retained
Was a model used to predict lipid molecule separation?	No	Lipid Identification Software	Shotgun Lipidomics Assistant (version not retained)
Nomenclature for intact lipid molecule	Yes	Nomenclature for fragment ions	N/A
Further identification remarks	Type II correction is assumed from the matched public SLA v1.3 workflow; study-specific settings are unknown. Standard product-ion verification is not applicable: FA uses Q1=Q3 pseudo-MRM of the intact ion. Ion forms and DMS assignments are public-workflow references (Sheet S8). Background checking denotes prepared-blank monitoring; interference criteria are unknown.		

4) FA[M-H]- / Lipid quantification

Quantitative	Yes	MS Level for quantification	MS^2

Internal lipid standard(s) MS^2

Internal standard	Fragment(s)	Endogenous subclass
1 class-matched isotope-labeled standard; see Supplemental Sheet S9	No fragment (Q1=Q3 pseudo-MRM)	Analyte-to-standard assignment not retained

Type of quantification	Internal standard amount	Response correction	No
Type I isotope correction	Yes	Limit of quantification	Unknown; study-specific LOQ criteria were not retained.
Normalization to reference	Yes	Lipid Quantification Software	Shotgun Lipidomics Assistant (version not retained)
Batch correction	Original batch correction is unknown. No further batch correction was applied to the supplied concentrations during revision.	Further quantification remarks	Internal-standard quantification, plasma-volume normalization and Type I correction are inferred from the matched SLA workflow. No separate species-response model is assumed beyond internal-standard normalization. Original settings and analyte-to-standard assignments are unknown. No additional correction of supplied concentrations was applied during revision.

5) HexCer[M+H]+ / Lipid identification

Lipid class	HexCer	MS Level for identification	MS^2
Identification level	Molecular species level	MS^2 adduct	[M+H]+

Fragments for identification

Fragment name
Product-ion m/z; named fragment not retained (see Supplemental Sheet S8)

Isotope correction at MS^2	Type 2	MS^2 verified by standard	No
Background check at MS^2	Yes	Did you presume assumptions for identification?	Yes
Which assumptions were presumed?	Assignments follow the predefined MRM panel at the reported structural level; positional isomers are not established.	Limit of detection	Unknown; study-specific LOD criteria were not retained.
Additional dimension/techniques	SelexION differential mobility spectrometry (DMS)	How was/were the additional dimension(s) used?	Public platform reference: DMS transparent/off; study-specific settings were not retained
Was a model used to predict lipid molecule separation?	No	Lipid Identification Software	Shotgun Lipidomics Assistant (version not retained)
Nomenclature for intact lipid molecule	Yes	Nomenclature for fragment ions	N/A

Further identification remarks	Type II correction is assumed from the matched public SLA v1.3 workflow; study-specific settings are unknown. No same-class standard is listed; the No verification selection is provisional because separate product-ion checking is unknown. Ion forms and DMS assignments are public-workflow references (Sheet S8). Background checking denotes prepared-blank monitoring; interference criteria are unknown.

5) HexCer[M+H]+ / Lipid quantification

Quantitative	Yes	MS Level for quantification	MS^2

Internal lipid standard(s) MS^2

Internal standard	Fragment(s)	Endogenous subclass
No name-matched standard listed in Supplemental Sheet S9	Product-ion m/z; name not retained	Analyte-to-standard assignment not retained

Type of quantification	Internal standard amount	Response correction	No
Type I isotope correction	Yes	Limit of quantification	Unknown; study-specific LOQ criteria were not retained.
Normalization to reference	Yes	Lipid Quantification Software	Shotgun Lipidomics Assistant (version not retained)
Batch correction	Original batch correction is unknown. No further batch correction was applied to the supplied concentrations during revision.	Further quantification remarks	Internal-standard quantification, plasma-volume normalization and Type I correction are inferred from the matched SLA workflow. No separate species-response model is assumed beyond internal-standard normalization. Original settings and analyte-to-standard assignments are unknown. No additional correction of supplied concentrations was applied during revision.

6) LPC[M+H]+ / Lipid identification

Lipid class	LPC	MS Level for identification	MS^2
Identification level	Species level	MS^2 adduct	[M+H]+

Fragments for identification

Fragment name
Product-ion m/z; named fragment not retained (see Supplemental Sheet S8)

Isotope correction at MS^2	Type 2	MS^2 verified by standard	Yes
Background check at MS^2	Yes	Did you presume assumptions for identification?	Yes
Which assumptions were presumed?	Assignments follow the predefined MRM panel at the reported structural level; positional isomers are not established.	Limit of detection	Unknown; study-specific LOD criteria were not retained.

Additional dimension/techniques	SelexION differential mobility spectrometry (DMS)	How was/were the additional dimension(s) used?	Public platform reference: DMS transparent/off; study-specific settings were not retained
Was a model used to predict lipid molecule separation?	No	Lipid Identification Software	Shotgun Lipidomics Assistant (version not retained)
Nomenclature for intact lipid molecule	Yes	Nomenclature for fragment ions	N/A
Further identification remarks	Type II correction is assumed from the matched public SLA v1.3 workflow; study-specific settings are unknown. Class-level product-ion checking is inferred from the listed isotope standards; an independent verification experiment is not documented. Ion forms and DMS assignments are public-workflow references (Sheet S8). Background checking denotes prepared-blank monitoring; interference criteria are unknown.		

6) LPC[M+H]+ / Lipid quantification

Quantitative	Yes	MS Level for quantification	MS^2

Internal lipid standard(s) MS^2

Internal standard	Fragment(s)	Endogenous subclass
3 class-matched isotope-labeled standards; see Supplemental Sheet S9	Product-ion m/z; name not retained	Analyte-to-standard assignment not retained

Type of quantification	Internal standard amount	Response correction	No
Type I isotope correction	Yes	Limit of quantification	Unknown; study-specific LOQ criteria were not retained.
Normalization to reference	Yes	Lipid Quantification Software	Shotgun Lipidomics Assistant (version not retained)
Batch correction	Original batch correction is unknown. No further batch correction was applied to the supplied concentrations during revision.	Further quantification remarks	Internal-standard quantification, plasma-volume normalization and Type I correction are inferred from the matched SLA workflow. No separate species-response model is assumed beyond internal-standard normalization. Original settings and analyte-to-standard assignments are unknown. No additional correction of supplied concentrations was applied during revision.

7) LPE[M+H]+ / Lipid identification

Lipid class	LPE	MS Level for identification	MS^2
Identification level	Species level	MS^2 adduct	[M+H]+

Fragments for identification

Fragment name
Product-ion m/z; named fragment not retained (see Supplemental Sheet S8)

Isotope correction at MS^2	Type 2	MS^2 verified by standard	Yes

Background check at MS^2	Yes	Did you presume assumptions for identification?	Yes
Which assumptions were presumed?	Assignments follow the predefined MRM panel at the reported structural level; positional isomers are not established.	Limit of detection	Unknown; study-specific LOD criteria were not retained.
Additional dimension/techniques	SelexION differential mobility spectrometry (DMS)	How was/were the additional dimension(s) used?	Public platform reference: DMS transparent/off; study-specific settings were not retained
Was a model used to predict lipid molecule separation?	No	Lipid Identification Software	Shotgun Lipidomics Assistant (version not retained)
Nomenclature for intact lipid molecule	Yes	Nomenclature for fragment ions	N/A
Further identification remarks	Type II correction is assumed from the matched public SLA v1.3 workflow; study-specific settings are unknown. Class-level product-ion checking is inferred from the listed isotope standards; an independent verification experiment is not documented. Ion forms and DMS assignments are public-workflow references (Sheet S8). Background checking denotes prepared-blank monitoring; interference criteria are unknown.		

7) LPE[M+H]+ / Lipid quantification

Quantitative	Yes	MS Level for quantification	MS^2

Internal lipid standard(s) MS^2

Internal standard	Fragment(s)	Endogenous subclass
3 class-matched isotope-labeled standards; see Supplemental Sheet S9	Product-ion m/z; name not retained	Analyte-to-standard assignment not retained

Type of quantification	Internal standard amount	Response correction	No
Type I isotope correction	Yes	Limit of quantification	Unknown; study-specific LOQ criteria were not retained.
Normalization to reference	Yes	Lipid Quantification Software	Shotgun Lipidomics Assistant (version not retained)
Batch correction	Original batch correction is unknown. No further batch correction was applied to the supplied concentrations during revision.	Further quantification remarks	Internal-standard quantification, plasma-volume normalization and Type I correction are inferred from the matched SLA workflow. No separate species-response model is assumed beyond internal-standard normalization. Original settings and analyte-to-standard assignments are unknown. No additional correction of supplied concentrations was applied during revision.

8) LPG[M+NH4]+ / Lipid identification

Lipid class	LPG	MS Level for identification	MS^2
Identification level	Species level	MS^2 adduct	[M+NH4]+

Fragments for identification

Fragment name
Product-ion m/z; named fragment not retained (see Supplemental Sheet S8)

Isotope correction at MS^2	Type 2	MS^2 verified by standard	Yes
Background check at MS^2	Yes	Did you presume assumptions for identification?	Yes
Which assumptions were presumed?	Assignments follow the predefined MRM panel at the reported structural level; positional isomers are not established.	Limit of detection	Unknown; study-specific LOD criteria were not retained.
Additional dimension/techniques	SelexION differential mobility spectrometry (DMS)	How was/were the additional dimension(s) used?	Public platform reference: DMS transparent/off; study-specific settings were not retained
Was a model used to predict lipid molecule separation?	No	Lipid Identification Software	Shotgun Lipidomics Assistant (version not retained)
Nomenclature for intact lipid molecule	Yes	Nomenclature for fragment ions	N/A
Further identification remarks	Type II correction is assumed from the matched public SLA v1.3 workflow; study-specific settings are unknown. Class-level product-ion checking is inferred from the listed isotope standards; an independent verification experiment is not documented. Ion forms and DMS assignments are public-workflow references (Sheet S8). Background checking denotes prepared-blank monitoring; interference criteria are unknown.		

8) LPG[M+NH4]+ / Lipid quantification

Quantitative	Yes	MS Level for quantification	MS^2

Internal lipid standard(s) MS^2

Internal standard	Fragment(s)	Endogenous subclass
3 class-matched isotope-labeled standards; see Supplemental Sheet S9	Product-ion m/z; name not retained	Analyte-to-standard assignment not retained

Type of quantification	Internal standard amount	Response correction	No
Type I isotope correction	Yes	Limit of quantification	Unknown; study-specific LOQ criteria were not retained.
Normalization to reference	Yes	Lipid Quantification Software	Shotgun Lipidomics Assistant (version not retained)
Batch correction	Original batch correction is unknown. No further batch correction was applied to the supplied concentrations during revision.	Further quantification remarks	Internal-standard quantification, plasma-volume normalization and Type I correction are inferred from the matched SLA workflow. No separate species-response model is assumed beyond internal-standard normalization. Original settings and analyte-to-standard assignments are unknown. No additional correction of supplied concentrations was applied during revision.

9) LPI[M+NH4]+ / Lipid identification

Lipid class	LPI	MS Level for identification	MS^2
Identification level	Species level	MS^2 adduct	[M+NH4]+

Fragments for identification

Fragment name
Product-ion m/z; named fragment not retained (see Supplemental Sheet S8)

Isotope correction at MS^2	Type 2	MS^2 verified by standard	Yes
Background check at MS^2	Yes	Did you presume assumptions for identification?	Yes
Which assumptions were presumed?	Assignments follow the predefined MRM panel at the reported structural level; positional isomers are not established.	Limit of detection	Unknown; study-specific LOD criteria were not retained.
Additional dimension/techniques	SelexION differential mobility spectrometry (DMS)	How was/were the additional dimension(s) used?	Public platform reference: DMS transparent/off; study-specific settings were not retained
Was a model used to predict lipid molecule separation?	No	Lipid Identification Software	Shotgun Lipidomics Assistant (version not retained)
Nomenclature for intact lipid molecule	Yes	Nomenclature for fragment ions	N/A
Further identification remarks	Type II correction is assumed from the matched public SLA v1.3 workflow; study-specific settings are unknown. Class-level product-ion checking is inferred from the listed isotope standards; an independent verification experiment is not documented. Ion forms and DMS assignments are public-workflow references (Sheet S8). Background checking denotes prepared-blank monitoring; interference criteria are unknown.		

9) LPI[M+NH4]+ / Lipid quantification

Quantitative	Yes	MS Level for quantification	MS^2

Internal lipid standard(s) MS^2

Internal standard	Fragment(s)	Endogenous subclass
3 class-matched isotope-labeled standards; see Supplemental Sheet S9	Product-ion m/z; name not retained	Analyte-to-standard assignment not retained

Type of quantification	Internal standard amount	Response correction	No
Type I isotope correction	Yes	Limit of quantification	Unknown; study-specific LOQ criteria were not retained.
Normalization to reference	Yes	Lipid Quantification Software	Shotgun Lipidomics Assistant (version not retained)

Batch correction	Original batch correction is unknown. No further batch correction was applied to the supplied concentrations during revision.	Further quantification remarks	Internal-standard quantification, plasma-volume normalization and Type I correction are inferred from the matched SLA workflow. No separate species-response model is assumed beyond internal-standard normalization. Original settings and analyte-to-standard assignments are unknown. No additional correction of supplied concentrations was applied during revision.

10) LPS[M+H]+ / Lipid identification

Lipid class	LPS	MS Level for identification	MS^2
Identification level	Species level	MS^2 adduct	[M+H]+
Fragments for identification			

Fragment name
Product-ion m/z; named fragment not retained (see Supplemental Sheet S8)

Isotope correction at MS^2	Type 2	MS^2 verified by standard	Yes
Background check at MS^2	Yes	Did you presume assumptions for identification?	Yes
Which assumptions were presumed?	Assignments follow the predefined MRM panel at the reported structural level; positional isomers are not established.	Limit of detection	Unknown; study-specific LOD criteria were not retained.
Additional dimension/techniques	SelexION differential mobility spectrometry (DMS)	How was/were the additional dimension(s) used?	Public platform reference: DMS transparent/off; study-specific settings were not retained
Was a model used to predict lipid molecule separation?	No	Lipid Identification Software	Shotgun Lipidomics Assistant (version not retained)
Nomenclature for intact lipid molecule	Yes	Nomenclature for fragment ions	N/A
Further identification remarks	Type II correction is assumed from the matched public SLA v1.3 workflow; study-specific settings are unknown. Class-level product-ion checking is inferred from the listed isotope standards; an independent verification experiment is not documented. Ion forms and DMS assignments are public-workflow references (Sheet S8). Background checking denotes prepared-blank monitoring; interference criteria are unknown.		

10) LPS[M+H]+ / Lipid quantification

Quantitative	Yes	MS Level for quantification	MS^2
Internal lipid standard(s) MS^2			

Internal standard	Fragment(s)	Endogenous subclass

3 class-matched isotope-labeled standards; see Supplemental Sheet S9	Product-ion m/z; name not retained	Analyte-to-standard assignment not retained

Type of quantification	Internal standard amount	Response correction	No
Type I isotope correction	Yes	Limit of quantification	Unknown; study-specific LOQ criteria were not retained.
Normalization to reference	Yes	Lipid Quantification Software	Shotgun Lipidomics Assistant (version not retained)
Batch correction	Original batch correction is unknown. No further batch correction was applied to the supplied concentrations during revision.	Further quantification remarks	Internal-standard quantification, plasma-volume normalization and Type I correction are inferred from the matched SLA workflow. No separate species-response model is assumed beyond internal-standard normalization. Original settings and analyte-to-standard assignments are unknown. No additional correction of supplied concentrations was applied during revision.

11) LacCer[M+H]+ / Lipid identification

Lipid class	LacCer	MS Level for identification	MS^2
Identification level	Molecular species level	MS^2 adduct	[M+H]+

Fragments for identification

Fragment name
Product-ion m/z; named fragment not retained (see Supplemental Sheet S8)

Isotope correction at MS^2	Type 2	MS^2 verified by standard	No
Background check at MS^2	Yes	Did you presume assumptions for identification?	Yes
Which assumptions were presumed?	Assignments follow the predefined MRM panel at the reported structural level; positional isomers are not established.	Limit of detection	Unknown; study-specific LOD criteria were not retained.
Additional dimension/techniques	SelexION differential mobility spectrometry (DMS)	How was/were the additional dimension(s) used?	Public platform reference: DMS transparent/off; study-specific settings were not retained
Was a model used to predict lipid molecule separation?	No	Lipid Identification Software	Shotgun Lipidomics Assistant (version not retained)
Nomenclature for intact lipid molecule	Yes	Nomenclature for fragment ions	N/A
Further identification remarks	Type II correction is assumed from the matched public SLA v1.3 workflow; study-specific settings are unknown. No same-class standard is listed; the No verification selection is provisional because separate product-ion checking is unknown. Ion forms and DMS assignments are public-workflow references (Sheet S8). Background checking denotes prepared-blank monitoring; interference criteria are unknown.		

11) LacCer[M+H]+ / Lipid quantification

Quantitative	Yes	MS Level for quantification	MS^2

Internal lipid standard(s) MS^2

Internal standard	Fragment(s)	Endogenous subclass
No name-matched standard listed in Supplemental Sheet S9	Product-ion m/z; name not retained	Analyte-to-standard assignment not retained

Type of quantification	Internal standard amount	Response correction	No
Type I isotope correction	Yes	Limit of quantification	Unknown; study-specific LOQ criteria were not retained.
Normalization to reference	Yes	Lipid Quantification Software	Shotgun Lipidomics Assistant (version not retained)
Batch correction	Original batch correction is unknown. No further batch correction was applied to the supplied concentrations during revision.	Further quantification remarks	Internal-standard quantification, plasma-volume normalization and Type I correction are inferred from the matched SLA workflow. No separate species-response model is assumed beyond internal-standard normalization. Original settings and analyte-to-standard assignments are unknown. No additional correction of supplied concentrations was applied during revision.

12) PA[M+NH4]+ / Lipid identification

Lipid class	PA	MS Level for identification	MS^2
Identification level	Species level	MS^2 adduct	[M+NH4]+

Fragments for identification

Fragment name
Product-ion m/z; named fragment not retained (see Supplemental Sheet S8)

Isotope correction at MS^2	Type 2	MS^2 verified by standard	No
Background check at MS^2	Yes	Did you presume assumptions for identification?	Yes
Which assumptions were presumed?	Assignments follow the predefined MRM panel at the reported structural level; positional isomers are not established.	Limit of detection	Unknown; study-specific LOD criteria were not retained.
Additional dimension/techniques	SelexION differential mobility spectrometry (DMS)	How was/were the additional dimension(s) used?	Public platform reference: DMS transparent/off; study-specific settings were not retained
Was a model used to predict lipid molecule separation?	No	Lipid Identification Software	Shotgun Lipidomics Assistant (version not retained)
Nomenclature for intact lipid molecule	Yes	Nomenclature for fragment ions	N/A

Further identification remarks	Type II correction is assumed from the matched public SLA v1.3 workflow; study-specific settings are unknown. No same-class standard is listed; the No verification selection is provisional because separate product-ion checking is unknown. Ion forms and DMS assignments are public-workflow references (Sheet S8). Background checking denotes prepared-blank monitoring; interference criteria are unknown.

12) PA[M+NH4]+ / Lipid quantification

Quantitative	Yes	MS Level for quantification	MS^2

Internal lipid standard(s) MS^2

Internal standard	Fragment(s)	Endogenous subclass
No name-matched standard listed in Supplemental Sheet S9	Product-ion m/z; name not retained	Analyte-to-standard assignment not retained

Type of quantification	Internal standard amount	Response correction	No
Type I isotope correction	Yes	Limit of quantification	Unknown; study-specific LOQ criteria were not retained.
Normalization to reference	Yes	Lipid Quantification Software	Shotgun Lipidomics Assistant (version not retained)
Batch correction	Original batch correction is unknown. No further batch correction was applied to the supplied concentrations during revision.	Further quantification remarks	Internal-standard quantification, plasma-volume normalization and Type I correction are inferred from the matched SLA workflow. No separate species-response model is assumed beyond internal-standard normalization. Original settings and analyte-to-standard assignments are unknown. No additional correction of supplied concentrations was applied during revision.

13) PC[M+CH3COO]- / Lipid identification

Lipid class	PC	MS Level for identification	MS^2
Identification level	Molecular species level	MS^2 adduct	[M+CH3COO]-

Fragments for identification

Fragment name
Product-ion m/z; named fragment not retained (see Supplemental Sheet S8)

Isotope correction at MS^2	Type 2	MS^2 verified by standard	Yes
Background check at MS^2	Yes	Did you presume assumptions for identification?	Yes
Which assumptions were presumed?	Assignments follow the predefined MRM panel at the reported structural level; positional isomers are not established.	Limit of detection	Unknown; study-specific LOD criteria were not retained.

Additional dimension/techniques	SelexION differential mobility spectrometry (DMS)	How was/were the additional dimension(s) used?	Public platform reference: DMS on; study-specific settings were not retained
Was a model used to predict lipid molecule separation?	No	Lipid Identification Software	Shotgun Lipidomics Assistant (version not retained)
Nomenclature for intact lipid molecule	Yes	Nomenclature for fragment ions	N/A
Further identification remarks	Type II correction is assumed from the matched public SLA v1.3 workflow; study-specific settings are unknown. Class-level product-ion checking is inferred from the listed isotope standards; an independent verification experiment is not documented. Ion forms and DMS assignments are public-workflow references (Sheet S8). Background checking denotes prepared-blank monitoring; interference criteria are unknown.		

13) PC[M+CH3COO]- / Lipid quantification

Quantitative	Yes	MS Level for quantification	MS^2

Internal lipid standard(s) MS^2

Internal standard	Fragment(s)	Endogenous subclass
5 class-matched isotope-labeled standards; see Supplemental Sheet S9	Product-ion m/z; name not retained	Analyte-to-standard assignment not retained

Type of quantification	Internal standard amount	Response correction	No
Type I isotope correction	Yes	Limit of quantification	Unknown; study-specific LOQ criteria were not retained.
Normalization to reference	Yes	Lipid Quantification Software	Shotgun Lipidomics Assistant (version not retained)
Batch correction	Original batch correction is unknown. No further batch correction was applied to the supplied concentrations during revision.	Further quantification remarks	Internal-standard quantification, plasma-volume normalization and Type I correction are inferred from the matched SLA workflow. No separate species-response model is assumed beyond internal-standard normalization. Original settings and analyte-to-standard assignments are unknown. No additional correction of supplied concentrations was applied during revision.

14) PE[M-H]- / Lipid identification

Lipid class	PE	MS Level for identification	MS^2
Identification level	Molecular species level	MS^2 adduct	[M-H]-

Fragments for identification

Fragment name
Product-ion m/z; named fragment not retained (see Supplemental Sheet S8)

Isotope correction at MS^2	Type 2	MS^2 verified by standard	Yes

Background check at MS^2	Yes	Did you presume assumptions for identification?	Yes
Which assumptions were presumed?	Assignments follow the predefined MRM panel at the reported structural level; positional isomers are not established.	Limit of detection	Unknown; study-specific LOD criteria were not retained.
Additional dimension/techniques	SelexION differential mobility spectrometry (DMS)	How was/were the additional dimension(s) used?	Public platform reference: DMS on; study-specific settings were not retained
Was a model used to predict lipid molecule separation?	No	Lipid Identification Software	Shotgun Lipidomics Assistant (version not retained)
Nomenclature for intact lipid molecule	Yes	Nomenclature for fragment ions	N/A
Further identification remarks	Type II correction is assumed from the matched public SLA v1.3 workflow; study-specific settings are unknown. Class-level product-ion checking is inferred from the listed isotope standards; an independent verification experiment is not documented. Ion forms and DMS assignments are public-workflow references (Sheet S8). Background checking denotes prepared-blank monitoring; interference criteria are unknown.		

14) PE[M-H]- / Lipid quantification

Quantitative	Yes	MS Level for quantification	MS^2

Internal lipid standard(s) MS^2

Internal standard	Fragment(s)	Endogenous subclass
5 class-matched isotope-labeled standards; see Supplemental Sheet S9	Product-ion m/z; name not retained	Analyte-to-standard assignment not retained

Type of quantification	Internal standard amount	Response correction	No
Type I isotope correction	Yes	Limit of quantification	Unknown; study-specific LOQ criteria were not retained.
Normalization to reference	Yes	Lipid Quantification Software	Shotgun Lipidomics Assistant (version not retained)
Batch correction	Original batch correction is unknown. No further batch correction was applied to the supplied concentrations during revision.	Further quantification remarks	Internal-standard quantification, plasma-volume normalization and Type I correction are inferred from the matched SLA workflow. No separate species-response model is assumed beyond internal-standard normalization. Original settings and analyte-to-standard assignments are unknown. No additional correction of supplied concentrations was applied during revision.

15) PG[M-H]- / Lipid identification

Lipid class	PG	MS Level for identification	MS^2
Identification level	Molecular species level	MS^2 adduct	[M-H]-

Fragments for identification

Fragment name
Product-ion m/z; named fragment not retained (see Supplemental Sheet S8)

Isotope correction at MS^2	Type 2	MS^2 verified by standard	Yes
Background check at MS^2	Yes	Did you presume assumptions for identification?	Yes
Which assumptions were presumed?	Assignments follow the predefined MRM panel at the reported structural level; positional isomers are not established.	Limit of detection	Unknown; study-specific LOD criteria were not retained.
Additional dimension/techniques	SelexION differential mobility spectrometry (DMS)	How was/were the additional dimension(s) used?	Public platform reference: DMS on; study-specific settings were not retained
Was a model used to predict lipid molecule separation?	No	Lipid Identification Software	Shotgun Lipidomics Assistant (version not retained)
Nomenclature for intact lipid molecule	Yes	Nomenclature for fragment ions	N/A
Further identification remarks	Type II correction is assumed from the matched public SLA v1.3 workflow; study-specific settings are unknown. Class-level product-ion checking is inferred from the listed isotope standards; an independent verification experiment is not documented. Ion forms and DMS assignments are public-workflow references (Sheet S8). Background checking denotes prepared-blank monitoring; interference criteria are unknown.		

15) PG[M-H]- / Lipid quantification

Quantitative	Yes	MS Level for quantification	MS^2

Internal lipid standard(s) MS^2

Internal standard	Fragment(s)	Endogenous subclass
4 class-matched isotope-labeled standards; see Supplemental Sheet S9	Product-ion m/z; name not retained	Analyte-to-standard assignment not retained

Type of quantification	Internal standard amount	Response correction	No
Type I isotope correction	Yes	Limit of quantification	Unknown; study-specific LOQ criteria were not retained.
Normalization to reference	Yes	Lipid Quantification Software	Shotgun Lipidomics Assistant (version not retained)
Batch correction	Original batch correction is unknown. No further batch correction was applied to the supplied concentrations during revision.	Further quantification remarks	Internal-standard quantification, plasma-volume normalization and Type I correction are inferred from the matched SLA workflow. No separate species-response model is assumed beyond internal-standard normalization. Original settings and analyte-to-standard assignments are unknown. No additional correction of supplied concentrations was applied during revision.

16) PI[M-H]- / Lipid identification

Lipid class	PI	MS Level for identification	MS^2
Identification level	Molecular species level	MS^2 adduct	[M-H]-

Fragments for identification

Fragment name
Product-ion m/z; named fragment not retained (see Supplemental Sheet S8)

Isotope correction at MS^2	Type 2	MS^2 verified by standard	Yes
Background check at MS^2	Yes	Did you presume assumptions for identification?	Yes
Which assumptions were presumed?	Assignments follow the predefined MRM panel at the reported structural level; positional isomers are not established.	Limit of detection	Unknown; study-specific LOD criteria were not retained.
Additional dimension/techniques	SelexION differential mobility spectrometry (DMS)	How was/were the additional dimension(s) used?	Public platform reference: DMS on; study-specific settings were not retained
Was a model used to predict lipid molecule separation?	No	Lipid Identification Software	Shotgun Lipidomics Assistant (version not retained)
Nomenclature for intact lipid molecule	Yes	Nomenclature for fragment ions	N/A
Further identification remarks	Type II correction is assumed from the matched public SLA v1.3 workflow; study-specific settings are unknown. Class-level product-ion checking is inferred from the listed isotope standards; an independent verification experiment is not documented. Ion forms and DMS assignments are public-workflow references (Sheet S8). Background checking denotes prepared-blank monitoring; interference criteria are unknown.		

16) PI[M-H]- / Lipid quantification

Quantitative	Yes	MS Level for quantification	MS^2

Internal lipid standard(s) MS^2

Internal standard	Fragment(s)	Endogenous subclass
5 class-matched isotope-labeled standards; see Supplemental Sheet S9	Product-ion m/z; name not retained	Analyte-to-standard assignment not retained

Type of quantification	Internal standard amount	Response correction	No
Type I isotope correction	Yes	Limit of quantification	Unknown; study-specific LOQ criteria were not retained.
Normalization to reference	Yes	Lipid Quantification Software	Shotgun Lipidomics Assistant (version not retained)

Batch correction	Original batch correction is unknown. No further batch correction was applied to the supplied concentrations during revision.	Further quantification remarks	Internal-standard quantification, plasma-volume normalization and Type I correction are inferred from the matched SLA workflow. No separate species-response model is assumed beyond internal-standard normalization. Original settings and analyte-to-standard assignments are unknown. No additional correction of supplied concentrations was applied during revision.

17) PS[M-H]- / Lipid identification

Lipid class	PS	MS Level for identification	MS^2
Identification level	Molecular species level	MS^2 adduct	[M-H]-

Fragments for identification

Fragment name
Product-ion m/z; named fragment not retained (see Supplemental Sheet S8)

Isotope correction at MS^2	Type 2	MS^2 verified by standard	Yes
Background check at MS^2	Yes	Did you presume assumptions for identification?	Yes
Which assumptions were presumed?	Assignments follow the predefined MRM panel at the reported structural level; positional isomers are not established.	Limit of detection	Unknown; study-specific LOD criteria were not retained.
Additional dimension/techniques	SelexION differential mobility spectrometry (DMS)	How was/were the additional dimension(s) used?	Public platform reference: DMS on; study-specific settings were not retained
Was a model used to predict lipid molecule separation?	No	Lipid Identification Software	Shotgun Lipidomics Assistant (version not retained)
Nomenclature for intact lipid molecule	Yes	Nomenclature for fragment ions	N/A
Further identification remarks	Type II correction is assumed from the matched public SLA v1.3 workflow; study-specific settings are unknown. Class-level product-ion checking is inferred from the listed isotope standards; an independent verification experiment is not documented. Ion forms and DMS assignments are public-workflow references (Sheet S8). Background checking denotes prepared-blank monitoring; interference criteria are unknown.		

17) PS[M-H]- / Lipid quantification

Quantitative	Yes	MS Level for quantification	MS^2

Internal lipid standard(s) MS^2

Internal standard	Fragment(s)	Endogenous subclass

5 class-matched isotope-labeled standards; see Supplemental Sheet S9	Product-ion m/z; name not retained	Analyte-to-standard assignment not retained

Type of quantification	Internal standard amount	Response correction	No
Type I isotope correction	Yes	Limit of quantification	Unknown; study-specific LOQ criteria were not retained.
Normalization to reference	Yes	Lipid Quantification Software	Shotgun Lipidomics Assistant (version not retained)
Batch correction	Original batch correction is unknown. No further batch correction was applied to the supplied concentrations during revision.	Further quantification remarks	Internal-standard quantification, plasma-volume normalization and Type I correction are inferred from the matched SLA workflow. No separate species-response model is assumed beyond internal-standard normalization. Original settings and analyte-to-standard assignments are unknown. No additional correction of supplied concentrations was applied during revision.

18) SM[M+H]+ / Lipid identification

Lipid class	SM	MS Level for identification	MS^2
Identification level	Molecular species level	MS^2 adduct	[M+H]+
Fragments for identification			

Fragment name
Product-ion m/z; named fragment not retained (see Supplemental Sheet S8)

Isotope correction at MS^2	Type 2	MS^2 verified by standard	Yes
Background check at MS^2	Yes	Did you presume assumptions for identification?	Yes
Which assumptions were presumed?	Assignments follow the predefined MRM panel at the reported structural level; positional isomers are not established.	Limit of detection	Unknown; study-specific LOD criteria were not retained.
Additional dimension/techniques	SelexION differential mobility spectrometry (DMS)	How was/were the additional dimension(s) used?	Public platform reference: DMS on; study-specific settings were not retained
Was a model used to predict lipid molecule separation?	No	Lipid Identification Software	Shotgun Lipidomics Assistant (version not retained)
Nomenclature for intact lipid molecule	Yes	Nomenclature for fragment ions	N/A
Further identification remarks	Type II correction is assumed from the matched public SLA v1.3 workflow; study-specific settings are unknown. Class-level product-ion checking is inferred from the listed isotope standards; an independent verification experiment is not documented. Ion forms and DMS assignments are public-workflow references (Sheet S8). Background checking denotes prepared-blank monitoring; interference criteria are unknown.		

18) SM[M+H]+ / Lipid quantification

Quantitative	Yes	MS Level for quantification	MS^2

Internal lipid standard(s) MS^2

Internal standard	Fragment(s)	Endogenous subclass
5 class-matched isotope-labeled standards; see Supplemental Sheet S9	Product-ion m/z; name not retained	Analyte-to-standard assignment not retained

Type of quantification	Internal standard amount	Response correction	No
Type I isotope correction	Yes	Limit of quantification	Unknown; study-specific LOQ criteria were not retained.
Normalization to reference	Yes	Lipid Quantification Software	Shotgun Lipidomics Assistant (version not retained)
Batch correction	Original batch correction is unknown. No further batch correction was applied to the supplied concentrations during revision.	Further quantification remarks	Internal-standard quantification, plasma-volume normalization and Type I correction are inferred from the matched SLA workflow. No separate species-response model is assumed beyond internal-standard normalization. Original settings and analyte-to-standard assignments are unknown. No additional correction of supplied concentrations was applied during revision.

19) TG[M+NH4]+ / Lipid identification

Lipid class	TG	MS Level for identification	MS^2
Identification level	Species level	MS^2 adduct	[M+NH4]+

Fragments for identification

Fragment name
Product-ion m/z; named fragment not retained (see Supplemental Sheet S8)

Isotope correction at MS^2	Type 2	MS^2 verified by standard	Yes
Background check at MS^2	Yes	Did you presume assumptions for identification?	Yes
Which assumptions were presumed?	Assignments follow the predefined MRM panel. TG annotations specify sum composition and one fatty-acyl fragment, not all three fatty-acyl chains or their positions.	Limit of detection	Unknown; study-specific LOD criteria were not retained.
Additional dimension/techniques	SelexION differential mobility spectrometry (DMS)	How was/were the additional dimension(s) used?	Public platform reference: DMS transparent/off; study-specific settings were not retained
Was a model used to predict lipid molecule separation?	No	Lipid Identification Software	Shotgun Lipidomics Assistant (version not retained)
Nomenclature for intact lipid molecule	No	Nomenclature for fragment ions	N/A

Further identification remarks	Type II correction is assumed from the matched public SLA v1.3 workflow; study-specific settings are unknown. Class-level product-ion checking is inferred from the listed isotope standards; an independent verification experiment is not documented. Ion forms and DMS assignments are public-workflow references (Sheet S8). Background checking denotes prepared-blank monitoring; interference criteria are unknown.

19) TG[M+NH4]+ / Lipid quantification

Quantitative	Yes	MS Level for quantification	MS^2

Internal lipid standard(s) MS^2

Internal standard	Fragment(s)	Endogenous subclass
9 class-matched isotope-labeled standards; see Supplemental Sheet S9	Product-ion m/z; name not retained	Analyte-to-standard assignment not retained

Type of quantification	Internal standard amount	Response correction	No
Type I isotope correction	Yes	Limit of quantification	Unknown; study-specific LOQ criteria were not retained.
Normalization to reference	Yes	Lipid Quantification Software	Shotgun Lipidomics Assistant (version not retained)
Batch correction	Original batch correction is unknown. No further batch correction was applied to the supplied concentrations during revision.	Further quantification remarks	Internal-standard quantification, plasma-volume normalization and Type I correction are inferred from the matched SLA workflow. No separate species-response model is assumed beyond internal-standard normalization. Original settings and analyte-to-standard assignments are unknown. No additional correction of supplied concentrations was applied during revision.
